# Fungal Genomics Challenges the Dogma of Name-Based Biosecurity

**DOI:** 10.1371/journal.ppat.1005475

**Published:** 2016-05-05

**Authors:** Alistair R. McTaggart, Magriet A. van der Nest, Emma T. Steenkamp, Jolanda Roux, Bernard Slippers, Louise S. Shuey, Michael J. Wingfield, André Drenth

**Affiliations:** 1 Department of Microbiology and Plant Pathology, Tree Protection Co-operative Programme (TPCP), Forestry and Agricultural Biotechnology Institute (FABI), University of Pretoria, Pretoria, Gauteng, South Africa; 2 Department of Genetics, Forestry and Agricultural Biotechnology Institute (FABI), University of Pretoria, Pretoria, Gauteng, South Africa; 3 Department of Plant Sciences, Tree Protection Co-operative Programme (TPCP), Forestry and Agricultural Biotechnology Institute (FABI), University of Pretoria, Pretoria, Gauteng, South Africa; 4 Queensland Alliance for Agriculture and Food Innovation (QAAFI), University of Queensland, Brisbane, Queensland, Australia; University of Pittsburgh, UNITED STATES

Microorganisms have inadvertently been spread via the global movement and trade of their substrates, such as animals, plants, and soil. This intercontinental exchange in the current era of globalisation has given rise to significant increases in the distribution of known pests and pathogens. Importantly, it has also resulted in many novel, emerging, infectious diseases. Biosecurity and quarantine, which aim to prevent the establishment of foreign or harmful organisms in a non-native area, are under significant pressure due to the massive increases in travel and trade.

Traditionally, quarantine regulations have been implemented based on pathogens that already cause significant disease problems on congener hosts in other parts of the world (e.g., Q-bank, available at http://www.q-bank.eu). Well-known pathogens are described, named, and studied to determine their disease cycle, epidemiology, and impact. Their importance is assessed based on their risk of infection, establishment, and economic or environmental consequences. This then shapes phytosanitary practices.

The central dogma of biosecurity proportions risk and focuses resources on known and named pathogens. This practice overlooks emerging pests and diseases that are increasingly spread around the world. Challenging this central dogma for biosecurity of fungi and fungal-like organisms is long overdue for the following reasons.

Firstly, in fungi, the rate of species discovery outpaces taxonomy, and naming of new taxa is not inherently accompanied by biological information. There are as many as three million (or more) fungal species in the world, and, of these, only 80,000 have been described [1]. Thus, a dogma that focuses on the described, well-known species overlooks the remainder and ignores their biosecurity significance. There are numerous examples of novel fungal and fungal-like pathogens that have caused major problems in environments with naïve hosts or monocultures in exotic locations. Some examples include *Cronartium ribicola* (white pine blister rust), *Ophiostoma novo-ulmi* (Dutch elm disease), *Phytophthora pinifolia*, and *Phytophthora ramorum* (sudden oak death).

Secondly, there is ongoing disagreement over the definition of species and use of taxonomic names. The dogma of biosecurity hinges on the application of a name, but this relies on a robust and accepted taxonomy. A recent example of this problem concerns an incursion of *Puccinia psidii* (rust of Myrtaceae), which was wrongly identified as a less severe, but closely related species, *Uredo rangelii*, in Australia in 2010. The incursion was downplayed, and a quarantine response stopped after nine days [2]. The objective of biosecurity, to safeguard biodiversity, was jeopardized by the current dogma and taxonomic politics, which questioned whether a species different from *P*. *psidii* would be as severe a threat. That this was an exotic pathogen with an undefined host range was neglected because of a name-based approach to biosecurity. *P*. *psidii* threatens native Australian species with extinction, and it will never be eradicated from Australia.

The third reason to reconsider a name-based dogma in biosecurity is that it does not account for genetic diversity in fungal populations. Invasion biology was traditionally concerned with the movement of taxa; however, the movement of intra- and interspecific novel genes or alleles is as important [3]. This is illustrated by genetic variants of *Puccinia graminis* and *Phytophthora infestans*, which caused dire consequences and new waves of disease in the cases of UG99 wheat stem rust and the introduction of a new mating type of late blight to Europe [4].

Fourthly, in addition to the introduction of new genetic strains or mating types of pathogens, fungi have very plastic genomes. They consequently exchange genetic material between species through hybridization and horizontal transfer of genes or entire chromosomes. For example, host specificity and pathogenicity of species of *Fusarium* are transferable by horizontal genetic exchange. This was demonstrated when a non-pathogenic strain of *Fusarium oxysporum* became pathogenic on tomato after horizontal transfer of an entire chromosome specific to a pathogenic strain [5]. Similar exchanges have been reported in other fungi, and this mechanism is suggested to have caused various plant disease epidemics in the 20th century [6].

Biosecurity, which is currently taxon-based, assesses risk for pathogens on quarantine lists. Obviously this excludes all pathogens that are not yet described or recognized. It also does not take into account the risks posed by the invasion of genes, transposons, or chromosomes into potential pathogens. The introduction of new fungal genes into a region can give rise to unforeseen consequences with regard to the development, pathogenicity, and aggressiveness of pathogen populations.

A paradigm shift is needed to overcome these serious shortcomings in biosecurity. Risk assessments should target the genes of pathogens rather than their names. Genomic research over the last decade has paved the way towards gene-based biosecurity. Detailed information about fungal genomes can help predict risks posed by undescribed pathogens through (*i*) **prediction of lifestyle**, e.g., biotrophic and saprotrophic fungi can be distinguished from nectrotrophic and hemibiotrophic fungi [7,8], and saprotrophic fungi can be distinguished from pathogens [7,9]. In time, protein families that exist in effective pathogens will be discovered and may be predictive for organisms that have an unknown ecology or life strategy. Software for rapid analysis of bacterial genomic data to screen for pathogenic proteins has been designed [10], and similar tools and databases will be developed for fungal pathogens. (*ii*) **Identification of potential pathogenicity factors**, i.e., factors necessary for disease development that suppress or manipulate host-cell physiology to the advantage of a pathogen, but which are not essential for a pathogen to complete its life cycle [8,11]. One example is disease effector proteins, which are likely expressed by all plant pathogens and may target similar defensive proteins in their hosts [12,13]. Effector genes do not have conserved motifs in fungi, and identifiers in the genome, such as diversifying selection, will be crucial to identify these genes that may be a clue to pathogenicity [14,15]. (*iii*) **Identification of transposable elements or high mutation rates**, which are implicated in the evolution of pathogenicity genes in fungi [7,9,16].

In the future, a wider, comprehensive approach to invasion biology must underpin risk assessment in biosecurity. It must assess risk in a more biologically meaningful manner. Likewise, a response to an incursion must consider characteristics that inform whether a fungus will be a risk, rather than focus merely on its name and the impact it might have caused elsewhere. Research focused on genomes, transcriptomes, proteomes, and metabolomes of fungi and fungal-like organisms is proliferating and, linked with biological information, can be used to determine whether an incursion poses a biosecurity risk.

We fully realize that paradigm shifts do not occur overnight, but the conversation amongst the scientific community urgently must grow in order to address this issue of global importance. This is an issue that will define the future of food security and the protection of the world’s natural biological resources.
